# Optimal timing of pharmacoinvasive strategy and its impact on clinical and economic outcomes in patients with ST-elevation myocardial infarction: a real-world perspective

**DOI:** 10.3389/fcvm.2024.1466961

**Published:** 2025-01-14

**Authors:** Andrea Dias Stephanus, Alice Pacheco Santos, Ana Carolina Machado Rodrigues da Cunha, Ana Carolina Augusto Rocha, Amanda de Amorim Meireles, Mariana Guimarães Souza de Oliveira, Pietra Arissa Coelho Matsunaga, Alexandre Anderson de Sousa Munhoz Soares, Ana Claudia Cavalcante Nogueira, Adriana de J. B. de Almeida Guimarães, Gustavo de Almeida Alexim, Alessandra M. Campos-Staffico, Luiz Sergio Fernandes de Carvalho

**Affiliations:** ^1^ Laboratory of Data for Quality of Care and Outcomes Research (LaDa:QCOR), Catholic University of Brasilia, Brasília, Brazil; ^2^ Aramari Apo Institute, Brasília, Brazil; ^3^ HEOR, Clarity Healthcare Intelligence, Jundiaí, Brazil; ^4^ Escola Superior de Ciências da Saúde (ESCS), Brasília, Brazil; ^5^ Secretaria de Estado de Saúde do Distrito Federal (SES-DF), Brasília, Brazil; ^6^ Department of Pharmacy Sciences | School of Pharmacy and Health Professions, Creighton University, Omaha, NE, United States

**Keywords:** ST-segment elevation myocardial infarction, fibrinolysis, percutaneous coronary intervention, pharmacoinvasive strategy, reperfusion strategy

## Abstract

**Background:**

The pharmacoinvasive (PhI) strategy is the standard-of-care for ST-elevation myocardial infarction (STEMI) patients when primary percutaneous coronary intervention (pPCI) is unfeasible. Optimal timing for post-fibrinolytic PCI (lysis-PCI) remains elusive. Therefore, this study aimed to assess the clinical and economic impacts of early vs. delayed lysis-PCI in patients with STEMI.

**Methods:**

This retrospective cohort study included 1,043 STEMI patients classified by lysis-PCI timing. The primary outcome was in-hospital major adverse cardiovascular events (4p-MACE), with secondary outcomes such as 3p-MACE, in-hospital mortality, and costs. Multivariable logistic regression models were used to assess the association between lysis-PCI timing and outcomes. Cost analyses were conducted from the perspective of Brazilian public healthcare system, with values converted to international dollars (Int$) for broader applicability.

**Results:**

Every 4-h delay in lysis-PCI was associated with a 44% reduction in in-hospital mortality [OR = 0.560 (95% CI: 0.381–0.775); *p* = 0.001] and a 13% decrease in 4p-MACE [OR = 0.877 (95% CI: 0.811–0.948); *p* = 0.001]. Additionally, 4-h delay in lysis-PCI was also associated with a significant reduction in in-hospital costs (Int$916.20 ± 99) and disease-induced years of productivity lost (*β* = −41.79 ± 151 years; *p* = 0.001). These significant trends remained consistent even after adjusting for confounders and applying propensity score matching. Older adults (aged ≥80) experienced an increase in 3p-MACE with earlier lysis-PCI.

**Conclusion:**

Delaying lysis-PCI was found to be associated with reduced in-hospital cardiovascular adverse events and lower costs, particularly among older adults. Further research should develop evidence-based lysis-PCI protocols that optimize both clinical outcomes and cost-effectiveness.

## 1 Introduction

Cardiovascular disease remains the preeminent global health threat, ending 39 lives every second as of 2022 (1). Within this domain, ischemic heart disease (IHD) ranks as the most lethal, accounting for nearly half of all cardiovascular deaths (1). Although advancements have been made in reducing the incidence of ST-segment elevation myocardial infarction (STEMI), the US still contends a stubbornly high mortality rate of 7.5% (2, 3), in stark contrast to the declining trends observed in non-STEMI deaths (4).

STEMI is the most time-sensitive acute coronary syndrome (5, 6), demanding rapid reperfusion interventions to save lives and mitigate disabilities (3). Evidence supports that prompt intervention significantly diminishes the severity of STEMI (7, 8), reduces mortality (8–10), and healthcare expenditures (10), highlighting the critical need for rapid restoration of coronary blood flow. Primary percutaneous coronary intervention (pPCI) within 90–120 minutes from the initial medical contact remains the gold standard treatment (11, 12). Despite improvements in pPCI access across the US (13), a substantial segment of the American population remains underserved (11). More than a quarter of STEMI cases are identified at hospitals that lack pPCI capabilities (11), and a majority of adult intensive care units are not equipped for post-procedure care (12). Alarmingly, two-thirds of US hospitals fail to meet the guideline-recommended time metrics, with 35% of transferred patients not achieving the vital 120-minute door-to-balloon benchmark (9).

In regions where pPCI is unfeasible, the pharmaco-invasive (PhI) strategy – characterized by post-fibrinolysis PCI within the first 24 hours – has been adopted as the standard of care for STEMI (6). This strategy is demonstrably superior to delayed elective post-fibrinolysis PCI, yielding reductions in cardiac mortality, reinfarction rates, and the need for further revascularization (11, 14).

Guidelines from leading cardiology associations recommend PCI timing post-fibrinolysis within windows that vary slightly: 2–24 hours according to the European Society of Cardiology, and 3–24 hours as per the American Heart Association (4, 6). Meta-analysis indicated that a 4-hour window post-fibrinolysis PCI is safe and optimal, though earlier interventions in aged adults increased the risk of bleeding, complications, and mortality (15). The most robust available evidence points to a limitation: the included clinical trials do not reflect the broader patient population, excluding those over 75, those with severe renal dysfunction, or those receiving PCI after 18 hours post-fibrinolysis. Thus, a definitive consensus on the safest and most effective timing for the PhI strategy remains elusive within the recommended 22-hours window.

The challenge of providing timely pPCI is compounded by the requirement for specialized facilities and highly skilled medical teams (16), which are not universally available, particularly in low- and middle-income countries (LMICs) (15). These regions, where over 75% of global cardiovascular deaths disproportionately occur (17, 18), place mounting pressure on healthcare systems. Although the PhI strategy has emerged as the primary treatment modality in LMICs, significant gaps remain in our understanding of the optimal timing for post-fibrinolysis PCI and its clinical and economic impacts (16). This study, therefore, aims to address this critical evidence gap by assessing the clinical and economic outcomes associated with varying intervals between fibrinolytic therapy administration and PCI initiation – commonly known as “lysis-PCI time” – among STEMI patients in a real-world PhI strategy setting.

## 2 Methods

### 2.2 Study design

This multicenter retrospective cohort study encompassed all patients diagnosed with STEMI who underwent a PhI strategy at two high-complexity public hospitals, namely *Hospital de Base do Distrito Federal* and *Instituto de Cardiologia do Distrito Federal*, in Brasília, Brazil, between January 2009 and December 2019. These facilities collectively managed 99.8% of public system catheterizations for acute coronary syndromes in the Brazilian capital.

### 2.3 Cohort identification

Participants were included if they: (i) were 18 years or older; (ii) had a confirmed electrocardiogram diagnosis of STEMI defined as ST-segment elevation of at least 1 mm (frontal plane) or 2 mm (horizontal plane) in 2 contiguous leads, or a newly presumed left bundle branch block or right bundle branch block; (iii) showed laboratory evidence of myocardial necrosis, indicated by an increase in at least one value above the 99th percentile reference limit of CK-MB (25 U/L) and troponin I (0.04 ng/mL), followed by a subsequent decline in both; (iv) received fibrinolytic therapy within 24 hours of symptom onset; (v) underwent PCI within 24 hours after fibrinolytic therapy. Patients who only received fibrinolytic therapy followed by rescue PCI were excluded. The patient selection process is outlined in Supplementary Figure S1.

Successful fibrinolysis was defined by clinical and electrocardiographic resolution, including significant chest pain relief, ≥50% ST-segment reduction in the lead with the highest elevation within 90 minutes post-fibrinolysis, and the absence of hemodynamic instability or persistent ischemic symptoms (19). Incorporating strict criteria for successful fibrinolysis ensures a homogeneous cohort, which is critical for isolating the effects of fibrinolysis-to-PCI timing on outcomes.

### 2.4 Ethics

The study protocol was approved by the local Institutional Review Board (IRB) (number CAAE 28530919.0.1001.8153), and a waiver of informed consent was granted, adhering to the ethical principles outlined in the Declaration of Helsinki.

### 2.5 Clinical and biochemical evaluation

Demographic, clinical, biochemical, and prescribed medications/therapies data were collected from electronic medical records by trained investigators using a standardized protocol. “Needle time”, the moment when fibrinolytic drugs were administered, was designated as the index time. Baseline characteristics were compiled up to one year prior to the index time.

### 2.6 Angiographic evaluation

Anatomical severity and the extent of coronary atherosclerotic disease, angiographic treatments, and left ventricular function were assessed through written reports. Severity of stenosis was classified as arterial lumen reduction >70% for epicardial vessels and >50% for the left main coronary artery. Multivessel disease was defined by the presence of >70% stenosis in three or more major epicardial vessels or >50% in the left main coronary artery. Left ventricular function was considered preserved if contractility was normal and classified as dysfunctional in the presence of hypokinesia or akinesia.

### 2.7 Study endpoints and adjudication of events

The primary endpoint was a composite of in-hospital major adverse cardiovascular events (4p-MACE), which included death, incident myocardial infarction, stroke, and heart failure symptoms arising over 24 hours post-STEMI. Secondary outcomes were: (i) 3p-MACE, comprising in-hospital death, recurrent myocardial infarction, ischemic stroke; (ii) in-hospital death alone; and (iii) cost outcomes including total, direct, and indirect in-hospital costs.

Death identification was conducted by consulting the Brazilian National Death Registry (SIM/SUS), which recorded causes of death through mandatory coding on death certificates.

### 2.8 Cost assessment

A comprehensive analysis of direct in-hospital costs, including expenses for procedures [e.g., PCI, coronary artery bypass graft surgery (CABG), intensive care unit (ICU) stays, and hemodialysis] was performed. Costs were evaluated from the perspective of the Brazilian Unified Health System (SUS) payer, with reimbursement based on standardized national rates from the SUS price list (details in Supplementary Table 1). Costs, denominated in Brazilian currency (R$), were converted to international dollars (Int$) using a purchasing power parity (PPP) conversion factor of 2.36. The methodology for extracting data from the SUS database has been previously described (18).

The indirect impact of STEMI on this population was evaluated using disease-induced years of productivity lost (DIYPL), a metric quantifying cumulative years of workforce participation lost due to health conditions, assuming retirement at 65 years (15). DIYPL provides insights into the societal impact of STEMI, capturing both disease-related productivity losses and premature mortality.

### 2.9 Statistical analysis

Study participants were categorized into three lysis-PCI time subgroups based on the tertiles of lysis-PCI time: ≤575 minutes (≤9.6 hours), 576–1,079 minutes (9.7–17.9 hours), and ≥1,080 minutes (≥18 hours).

Multiple imputation techniques were employed to handle missing values. Predictive mean matching model was used for numeric variables, logistic regression (logreg) for binary variables with two levels, and Bayesian polytomous regression (polyreg) for factor variables with two or more levels. The imputed values, residual distribution, and convergence coefficients were thoroughly checked. Missing values for the outcomes were not imputed.

Data distribution was evaluated using histograms, scatterplots, and normality tests (Kolmogorov-Smirnov or Shapiro-Wilk). Comparative statistical analysis varied based on the variable nature. Categorical variables were analyzed using chi-squared tests, while continuous variables were assessed using one-way analysis of variance (ANOVA) for normally distributed data or the Kruskal-Wallis test for non-normally distributed data. Normally distributed baseline data were expressed as means ± standard deviations, while non-normally distributed data were presented as medians [IQR] (interquartile ranges).

Logistic regression models assessed the association of lysis-PCI time tertiles with primary and secondary outcomes. Univariable regression models provided initial trends [odds ratios (OR), 95% confidence intervals (95%CI)]. Variables showing significant differences between subgroups at baseline were selected as covariates for multivariable adjustment. Additionally, a stepwise logistic multivariable regression model was used to identify key predictors of outcomes. Given the observational study design, propensity score matching (PSM) using a genetic matching algorithm (20–22) balanced baseline characteristics and weighted covariates between treatment (first tertile: ≤9.6 hours lysis-PCI) and control (second and third tertiles: > 9.6 hours lysis-PCI) groups. The Mahalanobis distance score and logistic regression machine learning identified matched pairs at a 1:1 ratio.

Age-specific analysis examined potential associations between lysis-PCI time and 30-day 4p-/3p-MACE odds using generalized linear models. An interaction term between age groups (<65, 65–80, and >80 years) and lysis-PCI time was included to assess whether the relationship between lysis-PCI time and MACE differed across age subgroups. Cubic splines were used to visualize the associations, allowing for a flexible, non-linear exploration of these interactions within each age group.

Statistical significance was defined as a *p*-value <0.05. Analyses were conducted using RStudio for Mac (version 1.1.463) with RGui language (version 4.0.1).

## 3 Results

### 3.1 Study population

A cohort of 1,043 patients diagnosed with STEMI undergoing a PhI strategy was established based on eligibility criteria. Patients were stratified into tertiles of lysis-PCI time: ≤9.6 hours (*n* = 344), 9.7–17.9 hours (*n* = 340), and ≥18 hours (*n* = 359). Overall, 56% of the cohort was male, and 64.7% were under the age of 65. Comprehensive baseline clinical and biochemical characteristics are presented in Table 1.

**Table 1:** Baseline clinical and biochemical characteristics of study patients.

	Overall	Lysis-PCI time			*p*-value
		≤9.6 hours	9.7–17.9 hours	≥18 hours	
Participants, *n* (%)	1,043 (100.0)	344 (33.0)	340 (32.6)	359 (34.4)	–
Male sex, *n* (%)	583 (55.9)	190 (55.2)	194 (57.1)	199 (55.4)	0.652
Age distribution, *n* (%)					0.311
<65 years	675 (64.7)	217 (63.1)	211 (62.1)	247 (68.8)	
65–79 years	320 (30.7)	108 (31.4)	114 (33.5)	98 (27.3)	
>80 years	48 (4.6)	19 (5.5)	15 (4.4)	14 (3.9)	
Comorbidities					
T2DM, *n* (%)	291 (27.9)	101 (29.4)	102 (30.0)	88 (24.5)	0.206
Obesity, *n* (%)	178 (17.1)	65 (18.9)	60 (17.7)	53 (14.8)	0.326
Alcohol consumption, *n* (%)	129 (12.4)	43 (12.5)	44 (12.9)	42 (11.7)	0.880
Smoking, *n* (%)	687 (65.9)	220 (64.0)	237 (69.7)	230 (64.1)	0.191
Hypertension, *n* (%)	595 (57.1)	203 (59.0)	192 (56.5)	200 (55.7)	0.654
Family history of CAD, *n* (%)	232 (22.2)	88 (25.6)	70 (20.6)	74 (20.6)	0.191
Drug abuse, *n* (%)	42 (4.0)	17 (4.9)	12 (3.5)	13 (3.6)	0.572
Dyslipidemia, *n* (%)	533 (51.1)	180 (52.3)	169 (49.7)	184 (51.3)	0.789
Hypothyroidism, *n* (%)	56 (5.4)	18 (5.2)	19 (5.6)	19 (5.3)	0.976
Prior AMI, *n* (%)	94 (9.0)	29 (8.4)	32 (9.4)	33 (9.2)	0.895
Prior stroke, *n* (%)	43 (4.1)	17 (4.9)	10 (2.9)	16 (4.5)	0.389
Prior PCI, *n* (%)	54 (5.2)	12 (3.5)	20 (5.9)	22 (6.1)	0.223
Prior PAD, *n* (%)	42 (4.0)	11 (3.2)	14 (4.1)	17 (4.7)	0.581
Prior CKD stage IV-V, *n* (%)	56 (5.4)	23 (6.7)	17 (5.0)	16 (4.5)	0.396
Prior CABG, *n* (%)	20 (1.9)	8 (2.3)	6 (1.8)	6 (1.7)	0.794
Prior AMI, PAD or stroke, *n* (%)	167 (16.0)	54 (15.7)	53 (15.6)	60 (16.7)	0.907
STEMI presentation					
Cardiac arrest at admission, *n* (%)	9 (0.9)	3 (0.9)	2 (0.6)	4 (1.1)	0.754
Right bundle branch block, *n* (%)	10 (1.0)	2 (0.6)	6 (1.8)	2 (0.6)	0.178
Left bundle branch block, *n* (%)	3 (0.3)	0 (0.0)	1 (0.3)	2 (0.6)	0.386
SBP at admission, mmHg	130.0 [32.0]	131.0 [31.5]	130.0 [26.0]	134.0 [37.0]	0.667
DBP at admission, mmHg	80.0 [21.0]	81.0 [21.0]	80.0 [20.0]	80.0 [24.0]	0.700
Killip classification at admission, *n* (%)					0.098
I	1,006 (96.5)	333 (96.8)	322 (94.7)	351 (97.8)	
II	36 (3.5)	11 (3.2)	18 (5.3)	7 (2.0)	
III	1 (0.1)	0 (0.0)	0 (0.0)	1 (0.3)	
IV	0 (0.0)	0 (0.0)	0 (0.0)	0 (0.0)	
Time intervals, minutes					
Pain to primary hospital	120.0 [148.0]	120.0 [140.0]	117.5 [140.0]	120.0 [160.0]	0.219
Door-lysis	70.0 [74.5]	62.5 [63.3]	75.0 [75.0]	73.0 [84.0]	**0**.**029**
Pain-lysis	215.0 [165.0]	210.0 [156.0]	215.0 [155.5]	220.0 [190.0]	0.081
Lysis-tertiary hospital admission	303.0 [286.0]	223.0 [166.0]	360.0 [363.5]	405.0 [409.0]	**<0**.**001**
Lysis-PCI	870.0 [739.0]	335.0 [191.5]	857.5 [245.0]	1,275.0 [185.0]	**<0**.**001**
Catheterization time length	50.0 [30.0]	45.0 [31.5]	50.0 [30.0]	50.0 [30.0]	0.124
PCI					
Individual average of conventional stents	0.84 ± 0.72	0.85 ± 0.65	0.86 ± 0.79	0.83 ± 0.71	0.918
Individual average of drug eluting stents	0.07 ± 0.30	0.04 ± 0.22	0.10 ± 0.36	0.07 ± 0.31	**0**.**027**
Glycoprotein IIb/IIIa inhibitors, *n* (%)	27 (2.6)	15 (4.4)	6 (1.8)	6 (1.7)	**0**.**041**
Angiographic findings					
1-vessel disease, *n* (%)	432 (41.4)	133 (38.7)	148 (43.5)	151 (42.1)	0.414
2-vessel disease, *n* (%)	323 (31.0)	110 (32.0)	103 (30.3)	110 (30.6)	0.881
3-vessel disease, *n* (%)	237 (22.7)	81 (23.5)	76 (22.4)	80 (22.3)	0.905
Left main disease, *n* (%)	9 (0.9)	4 (1.2)	2 (0.6)	3 (0.8)	0.717
No-reflow, *n* (%)	26 (2.5)	10 (2.9)	8 (2.4)	8 (2.2)	0.830
TIMI post-PCI, *n* (%)					0.228
0	144 (13.8)	42 (12.2)	58 (17.1)	44 (12.3)	
1	11 (1.1)	6 (1.8)	1 (0.3)	4 (1.1)	
2	127 (12.2)	43 (12.5)	37 (10.9)	47 (13.1)	
3	761 (73.0)	253 (73.6)	244 (71.8)	264 (73.5)	
Myocardial blush grade post-PCI, *n* (%)					0.235
0	191 (18.3)	69 (20.1)	52 (15.3)	70 (19.5)	
1	74 (7.1)	27 (7.9)	25 (7.4)	22 (6.1)	
2	50 (4.8)	22 (6.4)	16 (7.1)	12 (3.3)	
3	728 (69.8)	226 (65.7)	247 (72.7)	255 (71.0)	
Clinical scores and LVEF					
TIMI risk score	2.44 ± 1.05	2.47 ± 1.00	2.37 ± 1.13	2.48 ± 1.01	0.658
GRACE in-hospital death, points	106.0 [42.5]	107.0 [42.3]	108.0 [40.0]	104.0 [40.0]	0.200
CRUSADE, points	24.0 [19.0]	25.0 [18.0]	25.0 [20.0]	22.0 [19.0]	0.130
LVEF,%	47.0 [22.0]	45.5 [20.0]	46.5 [22.0]	49.0 [22.0]	0.501
Cardiac and biochemical biomarkers					
Peak troponin T, ng/L	5,769 [7,322]	6,868 [7,441]	5,825 [8,705]	4,860 [6,557]	**0**.**014**
Hemoglobin, g/L	14.5 [2.3]	14.7 [2.7]	14.4 [2.2]	14.6 [2.1]	0.431
Glycemia, mg/dL	118.0 [53.0]	116.0 [50.0]	122.0 [50.5]	116.0 [54.0]	0.074
HbA1c,%	5.7 [1.0]	5.7 [1.0]	5.7 [1.3]	5.6 [0.9]	**0**.**006**
TSH, mIU/mL	1.50 [1.59]	1.48 [1.60]	1.70 [1.75]	1.46 [1.58]	**0**.**037**
TC, mg/dL	199.0 [61.0]	199.0 [52.0]	200.0 [167.0]	199.0 [60.0]	0.998
HDL-C, mg/dL	41.0 [17.0]	41.0 [18.0]	42.0 [15.5]	40.0 [17.0]	0.791
LDL-C, mg/dL	123.0 [56.0]	121.0 [48.0]	131.0 [55.0]	117.0 [62.0]	**<0**.**001**
TG, mg/dL	129.0 [123.0]	128.0 [100.0]	129.0 [104.5]	132.0 [115.0]	0.098
Body mass index, kg/m^2^	26.1 [5.3]	26.0 [5.6]	26.0 [5.2]	26.2 [4.9]	0.962
Creatinine clearance, mL/min	91.0 [45.0]	90.0 [45.5]	88.0 [45.0]	93.0 [43.0]	0.446

T2DM, type 2 diabetes mellitus; CAD, coronary artery disease; AMI, acute myocardial infarction; PCI, percutaneous coronary intervention; PAD, peripheral artery disease; CKD, chronic kidney disease; CABG, coronary artery bypass graft surgery; AMI, acute myocardial infarction; SBP, systolic blood pressure; DBP, diastolic blood pressure; TIMI score, thrombolysis in myocardial infarction risk score; LVEF, left-ventricular ejection fraction; GRACE score, global registry of acute coronary events; CRUSADE, score for bleeding events; HbA1c, glycated hemoglobin; TSH, thyroid stimulating hormone; TC, total cholesterol; HDL-C, high-density lipoprotein cholesterol; LDL-C, low-density lipoprotein cholesterol; TG, triglycerides.

Bold values denote statistically significant results (*p* < 0.05).

Comparative statistical analysis revealed that patients in the ≤9.6 hours subgroup had significantly faster interventions, including shorter door-lysis and lysis-PCI durations, as well as quicker tertiary care transfers. Moreover, they more frequently received glycoprotein IIb/IIIa inhibitors and used drug-eluting stents less often. Conversely, the ≥18 hours subgroup had significantly lower peak levels of troponin T, HbA1c, TSH, and LDL-C. No other significant baseline differences were observed between the study subgroups (Table 1).

### 3.2 Clinical and cost outcomes

Further analysis identified differences in clinical and cost outcomes between the subgroups (Table 2). The ≤9.6 hours lysis-PCI window subgroup had significantly higher rates of in-hospital death, heart failure symptoms, left ventricular failure shock, cardiac arrest beyond the first 24 hours, major bleeding events, and 4p-MACE. This subgroup also incurred higher in-hospital costs and disease-related lost productivity years (Table 2).

**Table 2:** Comparison of clinical and cost outcomes among study subgroups.

	Lysis-PCI time			*p*-value
	≤9.6 hours	9.7–17.9 hours	≥18 hours	
Clinical outcomes				
In-hospital death, *n* (%)	12 (3.5)	5 (1.5)	1 (0.3)	**0**.**004**
Recurrent AMI, *n* (%)	5 (1.5)	5 (1.5)	4 (1.1)	0.898
Stroke, *n* (%)	2 (0.6)	3 (0.9)	5 (1.4)	0.536
CABG outcome, *n* (%)	15 (4.4)	16 (4.7)	13 (3.6)	0.757
Hospitalization length, days	5.03 ± 5.48	4.75 ± 6.33	4.55 ± 4.19	0.492
Heart failure symptoms after first 24 h, *n* (%)	135 (39.2)	119 (35.0)	97 (27.0)	**0**.**002**
Shock due LV failure after first 24 h, *n* (%)	14 (4.1)	7 (2.1)	0 (0.0)	**0**.**001**
Cardiac arrest after first 24 h, *n* (%)	20 (5.8)	9 (2.6)	5 (1.4)	**0**.**003**
Atrial fibrillation, *n* (%)	14 (4.1)	10 (2.9)	9 (2.5)	0.477
Major bleeding, *n* (%)	17 (4.9)	6 (1.8)	5 (1.4)	**0**.**006**
Blood transfusion, *n* (%)	6 (1.7)	2 (0.6)	2 (0.6)	0.189
3-p MACE, *n* (%)	17 (4.9)	13 (3.8)	10 (2.8)	0.330
4-p MACE, *n* (%)	137 (39.8)	122 (35.9)	98 (27.3)	**0**.**002**
Cost outcomes				
In-hospital costs, 1,000 Int$	32.3 [5.2]	31.1 [3.2]	29.4 [2.6]	**0**.**013**
DIYPL, years	457.3 ± 404.9	405.5 ± 379.9	293.8 ± 277.8	**<0**.**001**

AMI, acute myocardial infarction; CABG, coronary artery bypass graft surgery; LV, left ventricle; 3-p MACE, 3-point major adverse cardiovascular events (death, AMI, or stroke); 4-p MACE, 4-point major adverse cardiovascular events (death, recurrent AMI, stroke, or incident heart failure); DIYPL, disease-induced years of productivity lost.

Bold values denote statistically significant results (*p* < 0.05).

### 3.3 Propensity-matched cohort

To address the limitations of the observational study design concerning heterogeneity of baseline characteristics in the subgroups, we utilized PS-matching through a genetic algorithm. This method balanced baseline characteristics and weighted covariate between the treatment (first tertile: ≤9.6 hours lysis-PCI) and control (second and third tertiles: >9.6 hours lysis-PCI) subgroups. As shown in Supplementary Figure S2, PS-matching significantly improved the balance of both continuous and categorical covariates (Supplementary Table 2).

The PS-matching model included age, hypertension, type 2 diabetes, family history, angina, cocaine abuse, bundle branch blocks, door-to-needle time, and left anterior descending identification. The variables measured were drug-eluting stent count, presence of no-reflow, left ventricle ejection fraction (LVEF), admission heart rate, use of glycoprotein IIb/IIIa inhibitors, blood glucose, HbA1c, troponin, thyroid-stimulating hormone, and GRACE in-hospital mortality score.

In pairwise analysis, the treatment group showed an increased prevalence of prior stroke, while the control group exhibited more hypothyroidism, previous PCI, and elevated triglycerides. The control group also had longer lysis-to-hospital and lysis-to-PCI intervals, likely due these variables not being included in the PS model.

As expected, the control group experienced significantly longer door-lysis time and pain-to-lysis delays, slower transfers to tertiary hospitals post-lysis, and longer lysis-to-PCI procedures compared to the treatment group.

### 3.4 Association between lysis-PCI time and primary outcome

Three logistic regression models assessed the association between lysis-PCI time and 4p-MACE. The models included: (i) univariable (entire cohort); (ii) univariable (PS-matched cohort); and (iii) multivariable stepwise regression. Detailed results are shown in Table 3.

**Table 3:** Logistic regression models for in-hospital death (18 events), 3p-MACE (40 events), and 4p-MACE (357 events) as dependent variables.

Regression models	OR	95% CI		*p*-value
		Lower	Upper	
4p-MACE				
(Univariable model)				
Time from lysis to PCI (per 4-hour delay)	0.877	0.811	0.948	**0**.**001**
(Multivariable stepwise model)				
Time from lysis to PCI (per 4-hour delay)	0.890	0.801	0.951	**0**.**002**
LAD as the culprit artery	3.014	2.163	4.222	**<0**.**001**
Prior smoking	1.408	0.995	2.005	0.055
LV ejection fraction (per 10% decrease)	0.615	0.472	0.750	**<0**.**001**
No-reflow after PCI	5.575	1.851	18.368	**0**.**003**
Troponin peak (per 1,000 pg/mL increment)	1.044	1.024	1.064	**<0**.**001**
Glycemia at baseline (per 100 mg/dL increment)	1.518	1.250	1.791	**<0**.**001**
Hemoglobin at baseline (per 1 g/dL increment)	1.088	0.991	1.196	0.076
GRACE score for in-hospital death (per 10-point increase)	1.431	1.365	1.502	**<0**.**001**
(Propensity score-matched model)				
Time from lysis to PCI (per 4-hour delay)	0.929	0.867	0.985	**0**.**035**
3p-MACE				
(Univariable model)				
Time from lysis to PCI (per 4-hour delay)	0.856	0.705	0.973	**0**.**012**
(Multivariable stepwise model)				
Time from lysis to PCI (per 4-hour delay)	0.926	0.750	0.993	**0**.**046**
No-reflow after PCI	4.797	1.412	14.492	**0**.**008**
Glycemia at baseline (per 100 mg/dL increment)	1.566	1.152	1.960	**0**.**005**
GRACE score for in-hospital death (per 10-point increase)	1.351	1.251	1.459	**<0**.**001**
(Propensity score-matched model)				
Time from lysis to PCI (per 4-hour delay)	0.883	0.673	0.990	**0**.**045**
In-hospital death				
(Univariable model)				
Time from lysis to PCI (per 4-hour delay)	0.560	0.381	0.775	**0**.**001**
(Multivariable stepwise model)				
Time from lysis to PCI (per 4-hour delay)	0.597	0.401	0.837	**0**.**005**
GRACE score for in-hospital death (per 10-point increase)	1.461	1.321	1.620	**<0**.**001**
(Propensity score-matched model)				
Time from lysis to PCI (per 4-hour delay)	0.721	0.516	0.956	**0**.**034**
In-hospital costs (Int$)	**Beta**	**SD**		***p*-value**
(Univariable model)				
Time from lysis to PCI (per 4-hour delay)	−916.20	98.51		**<0**.**001**
(Propensity score-matched model)				
Time from lysis to PCI (per 4-hour delay)	−847.52	99.79		**0**.**007**

PCI, percutaneous coronary intervention; LAD, left anterior descending artery; LV, left ventricle; GRACE score, global registry of acute coronary events.

Bold values denote statistically significant results (*p* < 0.05).

Univariable analysis revealed a 12.3% reduction in 4p-MACE risk per 4-hour delay in lysis-PCI [OR: 0.877 (95% CI: 0.811–0.948); *p* = 0.001]. This relationship persisted in the multivariable stepwise regression and PS-matched analyses, with an 11% and 7.1% risk decrease per 4-hour delay, respectively (Table 3).

### 3.5 Association between lysis-PCI time and secondary outcomes

Every 4-hour lysis-PCI delay reduced the risk of 3p-MACE by 14.4% [OR: 0.856 (0.705–0.973); *p* = 0.012]. This remained significant across analyses, with an 11.7% decrease in 3p-MACE risk per 4-hour lysis-PCI delay in the PS-matched cohort (Table 3). Additionally, a 4-hour delay was associated with a 44.0% reduction in in-hospital death risk [OR: 0.560 (0.381–0.775); *p* = 0.001]. This association persisted across analyses, with a 27.9% decrease in in-hospital death risk per 4-hour delay in the PS-matched cohort (Table 3).

### 3.6 Cost analysis

The ≥18-hour lysis-PCI subgroup incurred significantly lower in-hospital costs [Int$ 29,400 (2,600); *p* = 0.013] and fewer disease-induced productivity loss years [293.8 ± 277.8 years; *p* < 0.001] compared to other subgroups (Table 2).

Every 4-hour lysis-PCI delay was associated with reduced productivity loss years (*β* −41.79 ± 150.55 years; *p* = 0.001) and in-hospital costs [Int$ −916.20 ± 98.51; *p* < 0.001]. PS-matched analysis remained consistent, with 4-hour lysis-PCI delays reducing productivity loss [*β* −31.35 ± 137.36 years; *p* = 0.008] and in-hospital costs [Int$ −847.5 ± 99.79; *p* = 0.007]. Both models showed an independent, inverse association between lysis-PCI time and disease burden and costs (Table 3; Supplementary Table 3).

### 3.7 Age-stratified sub-analysis on lysis-PCI time

**Figure 1:**
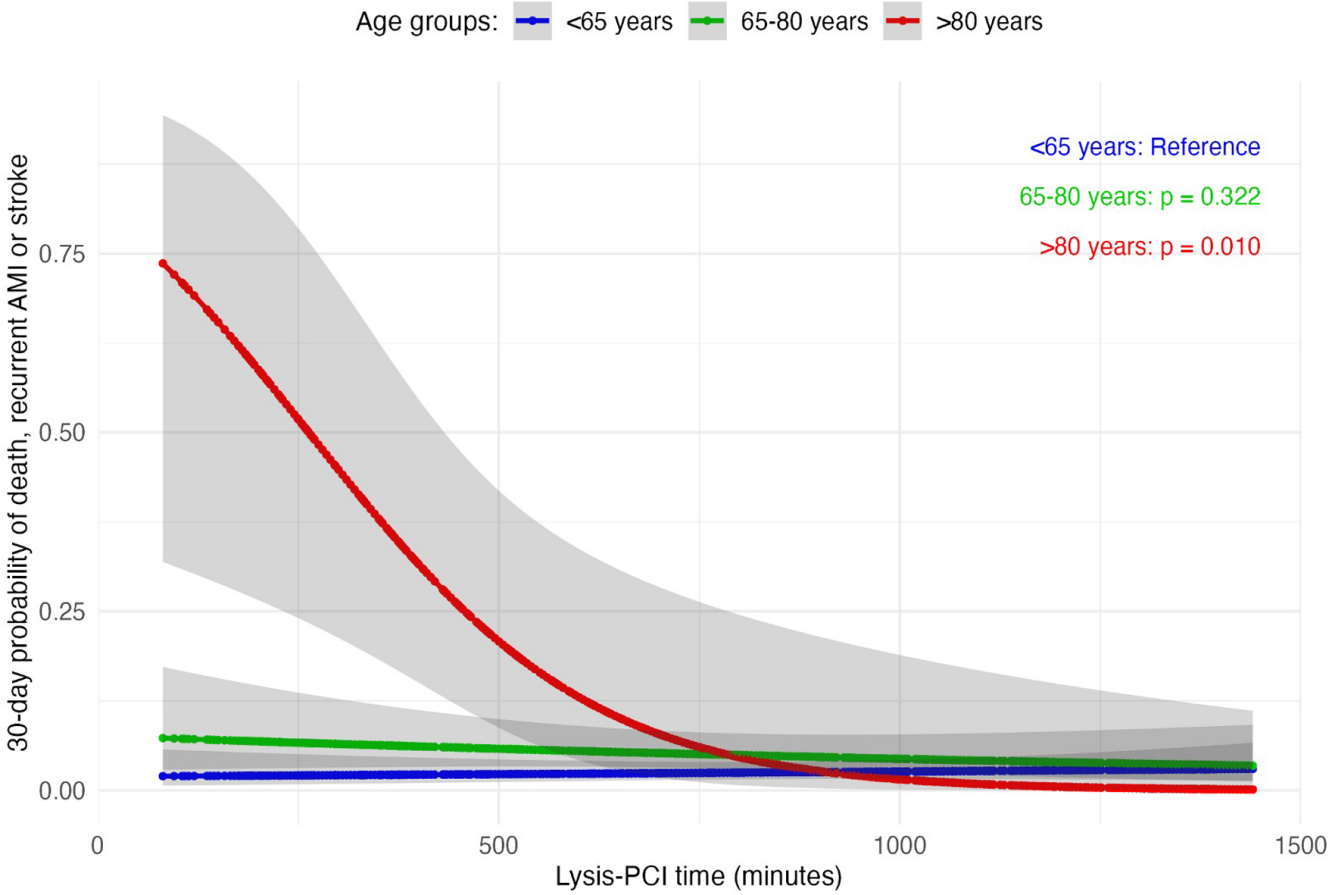
Generalized linear models of 30-day 3-point major adverse cardiovascular events (3p-MACE) probability distribution by lysis-PCI time across different age groups.

Having demonstrated a robust and consistent association between lysis-PCI time, outcomes, and costs, we examined variations in 30-day 3p-/4p-MACE probabilities across age groups using generalized linear models. Notably, STEMI patients over 80 years old exhibited a statistically significant higher 3p-MACE risk with early lysis-PCI (*p* = 0.010, Figure 1), with probabilities converging only beyond 500 min of lysis-PCI time, as evidenced by overlapping confidence intervals (Figure 1). Furthermore, early lysis-PCI in patients over 80 years showed an inverse trend with the 30-day 4p-MACE probability (Supplementary Figure S3), although this result did not reach statistical significance.

## 4 Discussion

To the best of our knowledge, this study is a pioneering effort in exploring real-world clinical and economic impacts of the fibrinolytic therapy-PCI timing gap for STEMI patients. Our research unveils robust, consistent evidence that ≤9.6-hour lysis-PCI is associated with increased risks of in-hospital mortality, 3p/4p-MACE, and elevated costs, regardless of the statistical approach employed. Specifically, every 4-hour lysis-PCI delay is associated with a 12.3% and 14.4% lower 4p- and 3p-MACE risk, respectively, and a substantial 44.0% reduction in in-hospital death risk.

Analysis of patients aged 80 years and older suggests that early lysis-PCI is associated with a higher 30-day probability of experiencing 3p-MACE, underscoring the need for personalized treatment strategies to mitigate risk in older STEMI populations. Our data also demonstrate that a ≤9.6-hour lysis-PCI is associated with greater direct and indirect costs. Importantly, these findings are consistent across age groups, with the relationship between lysis-PCI delays and reduced cardiovascular risk persisting even after excluding patients aged 80 years and older, known to be at higher risk for MACE (data not shown) (17, 23).

The heightened severity of symptoms observed in the ≤9.6-hour subgroup, including higher rates of in-hospital death, heart failure, cardiogenic shock, cardiac arrest, and major bleeding, supports the hypothesis that a shorter lysis-PCI interval may lead to more adverse outcomes and complications. This implies that the risks associated with rapid intervention might outweigh the potential benefits. These findings emphasize the importance of carefully considering the timing of PCI following fibrinolysis to optimize outcomes.

Although post-PCI TIMI 3 flow was slightly higher in the ≤9.6 hours group compared to the 9.7–17.9 hours and ≥18 hours groups, this observation was not accompanied by a proportional improvement in clinical outcomes. The myocardial blush grade (MBG), a key indicator of microvascular reperfusion and distal thrombotic burden (24), was worse in the ≤9.6 hours group, with a higher frequency of MBG 0 and 1. This discrepancy highlights the phenomenon of no-reflow, where the artery achieves patency (TIMI 3 flow) but microvascular obstruction limits effective perfusion (25). Furthermore, reduced contrast washout velocity, likely due to distal thrombi or microvascular damage, reinforces the disconnect between epicardial flow and myocardial reperfusion (26). These findings emphasize that achieving TIMI 3 flow does not guarantee improved prognosis, and that microvascular dysfunction plays a critical role in patient outcomes (27). While these trends were not statistically significant, they underscore the complexity of managing patients undergoing fibrinolysis followed by PCI and highlight the need for additional studies focusing on microcirculatory and inflammatory factors.

For STEMI management, prompt PCI is preferred due to its advantages over fibrinolytic therapy, such as superior infarct-artery patency, TIMI flow, and lower rates of complication like bleeding, ischemia, reinfarction, repeat revascularization, intracranial hemorrhage, and mortality (28). However, the accessibility of early PCI, which relies on specialized facilities and skilled teams, is limited, particularly in LMICs. For example, only 12.5% of Brazilian STEMI cases receive pPCI (29).

In contexts with limited resources, fibrinolytic therapy remains the first-line therapy for reducing STEMI mortality when administered within 8–12 hours of symptom onset (29). Combining fibrinolysis with routine early PCI improves outcomes where pPCI capabilities are lacking, although bleeding risks persist (30). Despite the growing adoption of pharmaco-invasive strategy, the optimal lysis-PCI interval for the best clinical and economic outcomes remains uncertain.

Our findings contest the prevailing view that minimizing myocardial ischemia through earlier reperfusion invariably improves outcomes (31). The mechanisms by which early lysis-PCI leads to increased MACE, mortality, and cost may involve heightened risks of major bleeding and no-reflow events. Our diverse cohort, which included about 5% of patients over 80 years and 5% with chronic kidney disease, highlights conditions known to increase post-fibrinolytic bleeding risk (23).

While our findings highlight the potential risks of early lysis-PCI, particularly in patients over 80 years old, it is important to emphasize that this study is observational and serves to generate hypotheses rather than provide definitive evidence to alter current guidelines or clinical practice. The small size of the 80+ subgroup, coupled with inherent survivorship bias due to their higher mortality rates, underscores the need for further investigation in larger cohorts. A larger sample would enable secondary analyses to explore additional clinical variables, such as renal dysfunction, that may influence outcomes and guide treatment personalization.

From a practical standpoint, our results do not suggest avoiding PCI within 4 hours following fibrinolysis but rather advocate for careful individual assessment of older and high-risk patients. In settings where primary PCI is unavailable, timely transfer to the catheterization lab remains essential, and further studies are needed to determine the optimal timing that balances clinical benefit and risk.

Advanced age significantly enhances the risk of no-reflow (30), characterized by microvascular thrombosis, distal embolization, and vasospasm, which are key predictors of short-term prognosis (32). Early combination therapy trials (31, 33–35) often exclude older patients due to their susceptibility to comorbidity-related event, limiting insights into adverse outcomes in this group. Consequently, the substantial representation of elderly patients in our cohort likely contributed to the observed increase in adverse outcomes associated with early lysis-PCI. Prompt post-fibrinolysis PCI may also have inadvertently worsened conditions in this vulnerable group.

While older patients face higher bleeding risks (17, 23), the increased bleeding in the ≤9.6-hour subgroup was not attributed to an overrepresentation of older adults, as the age distribution was not significantly different between subgroups. Furthermore, the relationship between lysis-PCI delays and reduced cardiovascular risk was consistent in a sensitivity analysis excluding patients aged 80 years and older (data not shown), reinforcing the reliability of our findings.

A randomized trial involving facilitated low-dose tenecteplase PCI, which specifically excluded participants aged ≥75 years (36), showed improved coronary artery patency and TIMI-3 flow rates (36). Consequently, the higher proportion of ≥80-year-olds in our study may have affected the increased early no-reflow observations.

Interestingly, a prior study indicated that matrix metalloproteinase 1 (MMP-1) peaks within 12 hours post-fibrinolysis among STEMI patients, coinciding with maximal tissue inhibitor of metalloproteinase-1 (TIMP-1) and MMP-1/TIMP-1 complex formation (37). This period of heightened MMP-1 activity without its natural inhibitor post-fibrinolysis (TIMP-1) may help explain the adverse outcome trends observed. MMP-1 inflammatory signaling responsiveness, combined with the heightened inflammatory milieu in older STEMI patients (37), suggests that plaque-disrupting PCI, an inflammatory intervention itself (38), might coincide with amplified harmful effects of MMP-1.

From a molecular standpoint, the data validates our hypothesis regarding the critical roles of matrix metalloproteinases (MMPs) in vascular remodeling and the instability of atherosclerotic plaques, mediated through inflammatory pathways (39). TIMP-1, the natural inhibitor, directly binds to MMP-1, affecting cardiac outcomes. Elevated MMP-1 levels in myocardial infarction patients, without corresponding increases in TIMP-1, correlate with poorer ventricular function (40). Furthermore, acute myocardial infarction reperfusion significantly alters MMP-1 levels over time (41).

A prior study demonstrated that after fibrinolysis in STEMI patients, MMP-1 levels rise, peaking with TIMP-1 between 9 and 12 hours post-fibrinolysis (37). This leads to the formation of the MMP-1/TIMP-1 complex. During the initial 12 hours post-fibrinolysis, increased MMP-1 activity due to inadequate TIMP-1 inhibition may contribute to adverse outcomes, including more frequent heart failure symptoms observed in patients with a lysis-PCI interval of ≤9.6 hours. Additionally, the heightened inflammatory response in older STEMI patients, coupled with the inflammatory nature of plaque-disrupting fibrinolysis, may intensify the harmful effects of MMP-1 (42). This could help explain why a shorter lysis-PCI interval, especially among older patients, could be particularly detrimental before the 12-hours peak of TIMP-1.

It is important to acknowledge certain limitation of our study. The data were derived from electronic medical records, which may have omitted key variables, leading to unmeasured residual confounding. Additionally, the study's retrospective and observational design introduces potential temporal bias and does not establish causality. However, the study was limited to two public hospitals that perform PCI procedures in the Brazilian capital, with minimal crossover between the public and private sectors. Although not a large multicenter trial, the uniqueness and consistency of our data and findings seem broadly relevant and plausible. However, limitations must be acknowledged, particularly the absence of documented PCI access routes. As femoral access increases bleeding risks compared to radial access (43), analyzing access distribution could have been informative. Unfortunately, this data was not available, making it impossible to determine whether increased bleeding in the ≤9.6-hours subgroup partly resulted from different access types – a limitation that precludes definitive conclusions regarding mechanisms. Ultimately, however, bleeding may relate at least in part to the shorter interval between fibrinolysis and PCI.

While our findings suggest potential risks of early lysis-PCI, particularly in patients over 80 years of age, we recognize that these results are hypothesis-generating and require confirmation in a robust, high-evidence study. Designing a randomized controlled trial (RCT) to test this hypothesis presents ethical and practical challenges, especially given the observational nature of our findings and the vulnerability of older patients. However, carefully constructed trials, with appropriate safeguards and patient selection criteria, are essential to determine the safest and most effective lysis-PCI timing, particularly in high-risk subgroups such as the elderly. Future studies are warranted to validate our results and inform clinical practice.

In conclusion, the lysis-PCI interval significantly impacts STEMI care. Our real-world analysis indicates that early PCI (≤9.6 hours) is associated with greater risks of mortality, MACE, and costs – particularly among the older adult. Further research should define the ideal fibrinolysis-PCI timing, balancing outcomes against costs to inform evidence-based guidelines.

## Data Availability

The datasets presented in this article are not readily available because the data contains sensitive medical information from a real-world clinical setting. The Institutional Review Board (IRB) that approved this study did not authorize the public disclosure of individual patient data to protect participant privacy. Requests to access these datasets should be directed to luizsergiofc@gmail.com; luiz.carvalho@p.ucb.br
