# Supplementary material for: Optimal timing of pharmacoinvasive strategy and its impact on clinical and economic outcomes in patients with ST-elevation myocardial infarction: a real-world perspective
Source: Front Cardiovasc Med. 2025 Jan 14;11:1466961. doi: 10.3389/fcvm.2024.1466961 (PMC11772263; doi:10.3389/fcvm.2024.1466961)

Supplementary Material

Supplementary Table 1. Average cost of cardiovascular procedures and hospitalizations in the Brazilian public healthcare system (SUS) between 2013 to 2015.

	**Average cost of CV procedures in the Brazilian Health System (SUS) (in Int$)**
Procedures typically performed in patients with STEMI (per 1 procedure)	
PCI	+6,328.00
CABG	+12,655.00
Intensive care unit (ICU) *hospitalization* (per day) *	555.00
In-patient care by condition treated	
Heart failure*	1,252.00
Cardiac arrest*	2,000.00
Myocardial infarction*	1,778.00
Ischemic stroke*	3,321.00
Haemorrhagic stroke**	7,043.00

CV: cardiovascular; **PCI:** percutaneous coronary intervention; **CABG**: coronary artery bypass graft surgery; **ICU:** Intensive care unit.

*The cost of hospitalization covers the expenses for laboratory and imaging examinations as well as bed fees during the average hospital stay length, but procedures are not included.

**Includes costs of procedures and hospitalization. Data for Brazil were obtained from DATASUS (SIH/SUS and SIGTAP), the data processing system of the Brazilian Ministry of Health.

We used the reimbursement costs for procedures, ICU admissions, and inpatient care to estimate the average annual costs associated with relevant clinical events (RECE), including hemodialysis, and their relative incidence in patient-years observed in B-CaRe:QCO.

Supplementary Table 2. Summarized data from genetic propensity-matching score with 728 pairs of patients treated with lysis-PCI within or after 9 hours.

	Lysis-PCI time		p-value
	≤9.6 h	>9.6 h	
**Patients, n (%)**	728 (50.0)	728 (50.0)	—
**Comorbidities**			
T2DM, n (%)	197 (27.1)	201 (27.6)	0.860
Obesity, n (%)	124 (17.0)	118 (16.2)	0.725
Alcohol consumption, n (%)	90 (12.4)	89 (12.2)	1.000
Smoking, n (%)	481 (66.1)	484 (66.5)	0.912
Hypertension, n (%)	425 (58.4)	409 (56.2)	0.427
Family history of CAD, n (%)	148 (20.3)	153 (21.0)	0.796
Drug abuse, n (%)	29 (4.0)	28 (3.8)	1.000
Dyslipidemia, n (%)	363 (49.9)	364 (50.0)	1.000
Hypothyroidism, n (%)	20 (2.7)	41 (5.6)	**0.009**
**Body mass index, kg/m²**	26.3 ± 4.3	26.6 ± 4.3	0.181
**Creatinine clearance, mL/min**	96.7 ± 35.5	94.1 ± 34.73	0.156
**Prior AMI, n (%)**	49 (6.7)	67 (9.2)	0.100
**Prior Stroke, n (%)**	63 (8.7)	26 (3.6)	**<0.001**
**Prior PCI, n (%)**	20 (2.7)	42 (5.8)	**0.006**
**Prior PAD, n (%)**	29 (4.0)	31 (4.3)	0.895
**Prior CKD, n (%)**	32 (4.4)	34 (4.7)	0.900
**Prior CABG, n (%)**	7 (1.0)	13 (1.8)	0.260
**STEMI presentation**			
SBP at admission	134.6 ± 23.2	134.0 ± 25.9	0.598
Killip classification at admission, n (%)			0.065
I	715 (98.2)	701 (96.3)	
II	13 (1.8)	26 (3.6)	
III	3 (0.9)	3 (0.9)	
IV	0 (0.0)	1 (0.1)	
**Time Intervals, minutes**			
Pain to Primary Hospital, minutes	145.2 ± 127.6	152.1 ± 122.6	0.287
Door-Lysis, minutes	64.00 [72.0]	75.00 [75.0]	**0.002**

Pain-Lysis, minutes	232.4 ± 138.3	250.2 ± 149.9	0.018
Lysis-Tertiary Hospital, minutes	234.7 ± 99.3	454.8 ± 302.1	**<0.001**
Lysis-PCI, minutes	330.9 ± 101.0	1044.8 ± 259.0	**<0.001**
Catheterization time length, minutes	58.5 ± 86.1	54.8 ± 25.0	0.263
**PCI**			
Individual average of conventional stents	0.85 ± 0.66	0.84 ± 0.75	0.809
Individual average of drug eluting stents	0.08 ± 0.31	0.08 ± 0.32	0.803
Glycoprotein IIb/IIIa inhibitors, n (%)	6 (0.8)	13 (1.8)	0.166
**Angiographic findings**			
No reflow, n (%)	16 (2.2)	16 (2.2)	1.000
TIMI post-PCI, n (%)			0.725
0	86 (11.8)	79 (10.9)	
1	7 (1.0)	5 (0.7)	
2	78 (10.7)	70 (9.6)	
3	557 (76.5)	574 (78.8)	
Myocardial blush grade post-PCI, n (%)			0.840
0	121 (16.6)	110 (15.1)	
1	52 (7.1)	49 (6.7)	
2	32 (4.4)	31 (4.3)	
3	523 (71.8)	538 (73.9)	
**Clinical scores and LV ejection fraction**			
GRACE in-hospital death, points	107.9 ± 29.3	110.0 ± 33.0	0.208
LV ejection fraction, %	47.0 ± 11.7	46.9 ± 13.2	0.878
**Cardiac Biomarkers**			
Troponin T at admission, ng/L	5762.5 [6260.0]	5385.0 [7390.3]	0.533
Hemoglobin, g/L	14.5 ± 1.5	14.5 ± 1.7	0.554
Glycemia, mg/dL	112.50 [40.0]	115.00 [44.0]	0.116
HbA1c, %	6.40 [6.60]	6.35 [5.66]	0.129
TSH, mIU/mL	1.37 [1.17]	1.41 [1.14]	0.523
HDL-C, mg/dL	39.1 ± 12.7	38.7 ± 13.5	0.504
LDL-C, mg/dL	113.9 ± 49.1	110.5 ± 52.2	0.209
TG, mg/dL	112.0 [84.0]	119.0 [102.0]	**0.017**
Creatinine, mg/dL	0.85 [0.30]	0.87 [0.28]	0.116

T2DM: type 2 diabetes mellitus; **CAD:** coronary artery disease; **AMI**: acute myocardial infarction; **PCI**: percutaneous coronary intervention; **PAD**: peripheral artery disease; **CKD**: chronic kidney disease; **CABG**: coronary artery bypass graft surgery; **AMI**: acute myocardial infarction; **SBP**: systolic blood pressure; **DBP**: diastolic blood pressure; **TIMI score**: thrombolysis in myocardial infarction risk score; **LVEF**: left-ventricular ejection fraction; **GRACE score**: global registry of acute coronary events; **CRUSADE**: score for bleeding events; **HbA1c**: glycated hemoglobin; **TSH**: thyroid stimulating hormone; **TC**: total cholesterol; **HDL-C**: high-density lipoprotein cholesterol; **LDL-C**: low-density lipoprotein cholesterol; **TG**: triglycerides.

Supplementary Table 3. Linear regression models for disease-induced years of productivity lost (DIYPL).

**DIYPL**	Beta	SD	p-value
(Bivariable model)			
Time from lysis to PCI (per 4-h delay)	-41.79	150.55	**<0.001**
(Multivariable stepwise model)			
Time from lysis to PCI (per 4-h delay)	-31.35	137.36	**0.008**

PCI: percutaneous coronary intervention.

Supplementary Figure S1. Flow chart with participants' selection.

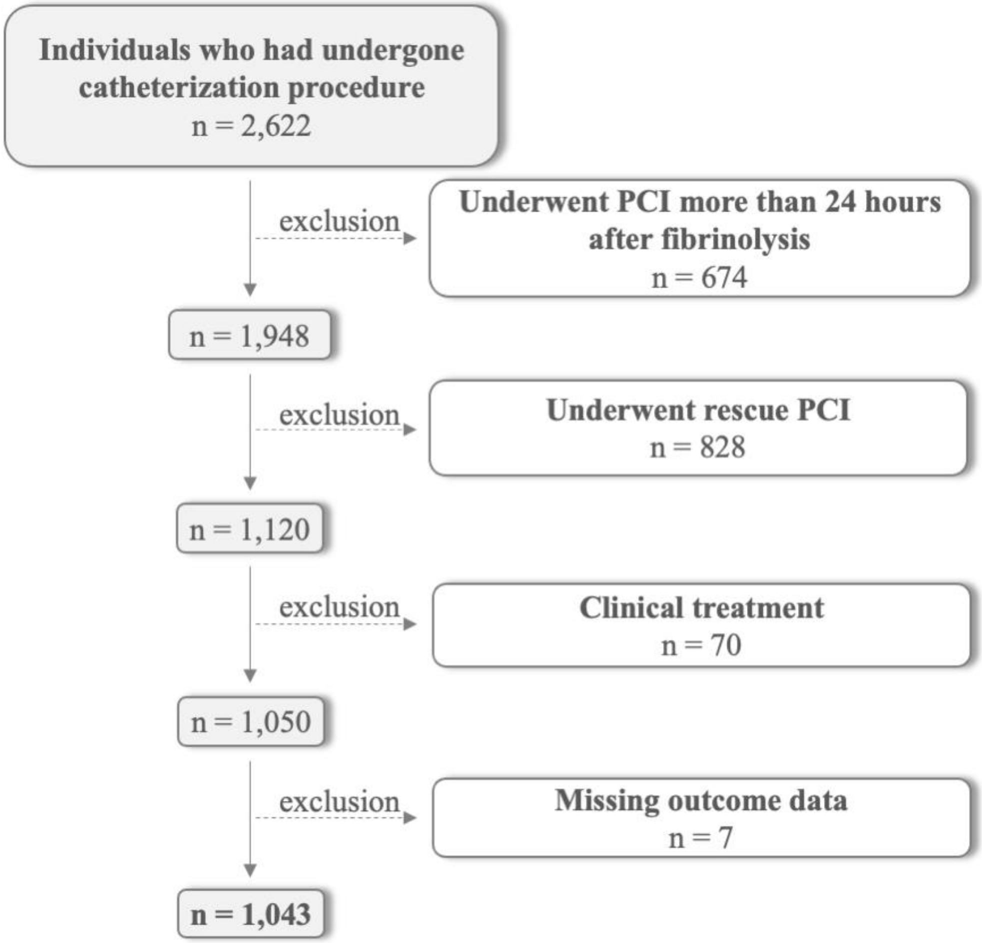

Supplementary Figure S2. Balance assessment before and after propensity score-matching. The group with a lysis-PCI time ≤9.6 h was designated as the “treatment”, while the group with a lysis-PCI time >9.6 h was designated as the “control” group.

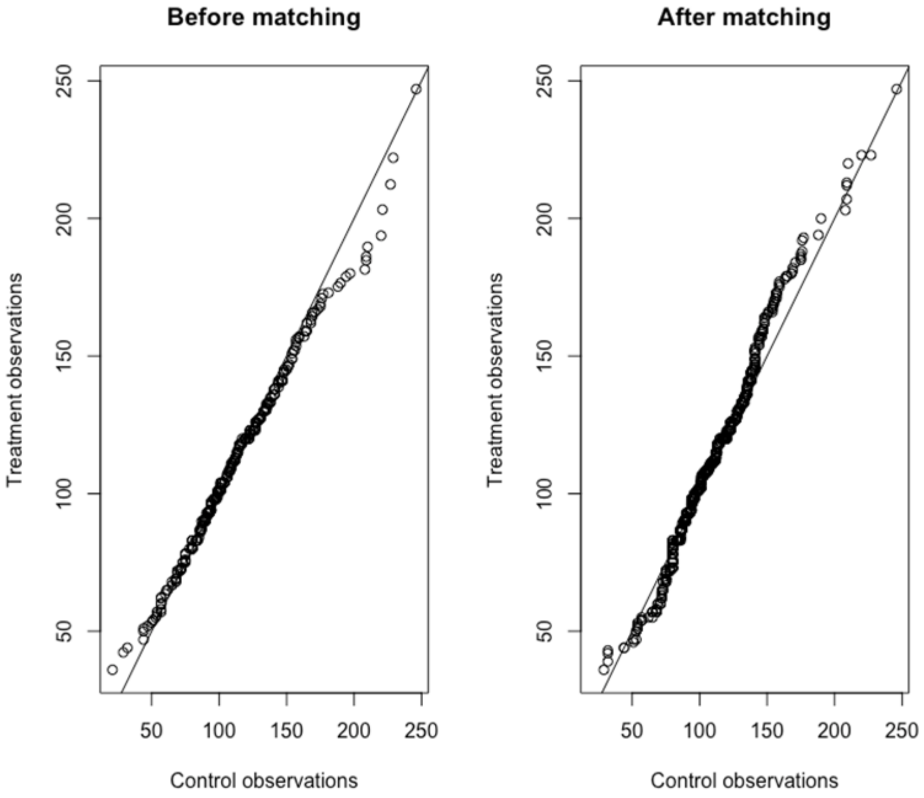

Supplementary Figure S3. Generalized linear models of 30-day 4-point major adverse cardiovascular events (4p-MACE) probability distribution by lysis-PCI time across different age groups.

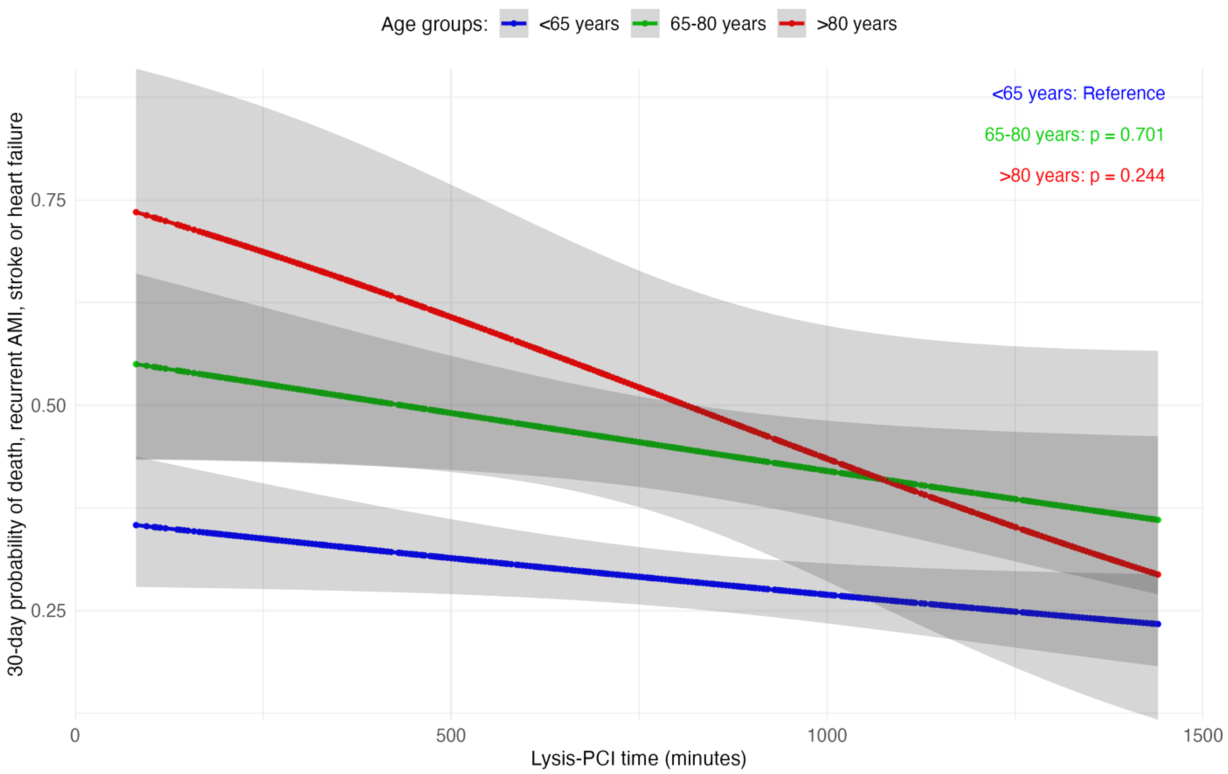

Supplement: Supplementary file 1 [file Datasheet1.pdf]
